# Cavernous Lymphangioma of the Neck: An Extraordinary Case

**DOI:** 10.7759/cureus.71075

**Published:** 2024-10-08

**Authors:** Mayur Ingale, Tharun Rajeev, Anvitha Suresh, Manu Babu, Gundappa Mahajan

**Affiliations:** 1 Otolaryngology-Head and Neck Surgery, Dr. D. Y. Patil Medical College, Hospital and Research Centre, Dr. D. Y. Patil Vidyapeeth, Pune, IND

**Keywords:** cavernous lymphangioma, cervical lymphangioma, neck swelling, submandibular gland surgery, surgical case report

## Abstract

Cervical lymphangioma is a congenital malformation of the lymphatic system that shows a predilection for the head and neck regions. They are most common in children between the ages of three and five and are rarely present in adults. While over 90% of lymphatic malformations are congenital, they can also manifest later due to factors such as trauma, infection, tumors, or medical procedures. They are often asymptomatic, but symptoms, if present, vary depending on the location of the tumor. Imaging techniques such as ultrasonography and computed tomography can aid in accurately mapping the extent and location of the lesion, thereby enhancing the effectiveness of the treatment. Complete surgical removal is regarded as the optimal approach to treatment. The following case presents a 35-year-old hypertensive female patient with swelling in the right side of the neck for the past seven months and diagnosed as a branchial cleft cyst with the help of ultrasonography and contrast-enhanced computed tomography of the neck. A complete surgical excision of the right submandibular gland was done, and a histopathological examination of the specimen revealed a cavernous lymphangioma. The unusual age group of presentation with no associated symptoms makes this case a rare find.

## Introduction

Lymphangioma of the neck is such a rare disease that only a few surgeons have the opportunity to study it directly. Apart from the rarity of the condition, it presents peculiar pathological and anatomical features that are not yet fully understood. They are most common in children between the ages of three and five and rarely present in adults [1-4]. While over 90% of lymphatic malformations are clearly congenital, they can also manifest later due to factors such as trauma, infection, tumors, or medical procedures. The pathophysiology of adult lymphangioma is not clearly understood but may occur secondary to inductions of dormant rest of embryonic lymphatic tissue that are stimulated to differentiate and grow [1]. These tumors possess the power of independent growth, and this growth occurs in the endothelial layer. The typical cavernous tumor may occur in the subcutaneous tissue or under the deep fascia. It consists of large anastomosing channels, which may contain in their midst irregular masses of lymphocytes, aberrant lymph follicles, and nodes [5]. They are often asymptomatic and can grow significantly before patients notice, sometimes not until adulthood. Symptoms, if present, vary depending on the location of the tumor; for instance, neck tumors present with swallowing difficulties or breathing problems, which require emergency surgery [6]. Imaging techniques such as ultrasonography and computed tomography can aid in accurately mapping the extent and location of the lesion, thereby enhancing the effectiveness of the treatment. Numerous therapeutic choices, like sclerotherapy, are documented in the literature; however, complete surgical removal is regarded as the optimal approach [1,3].

## Case presentation

**Figure 1:**
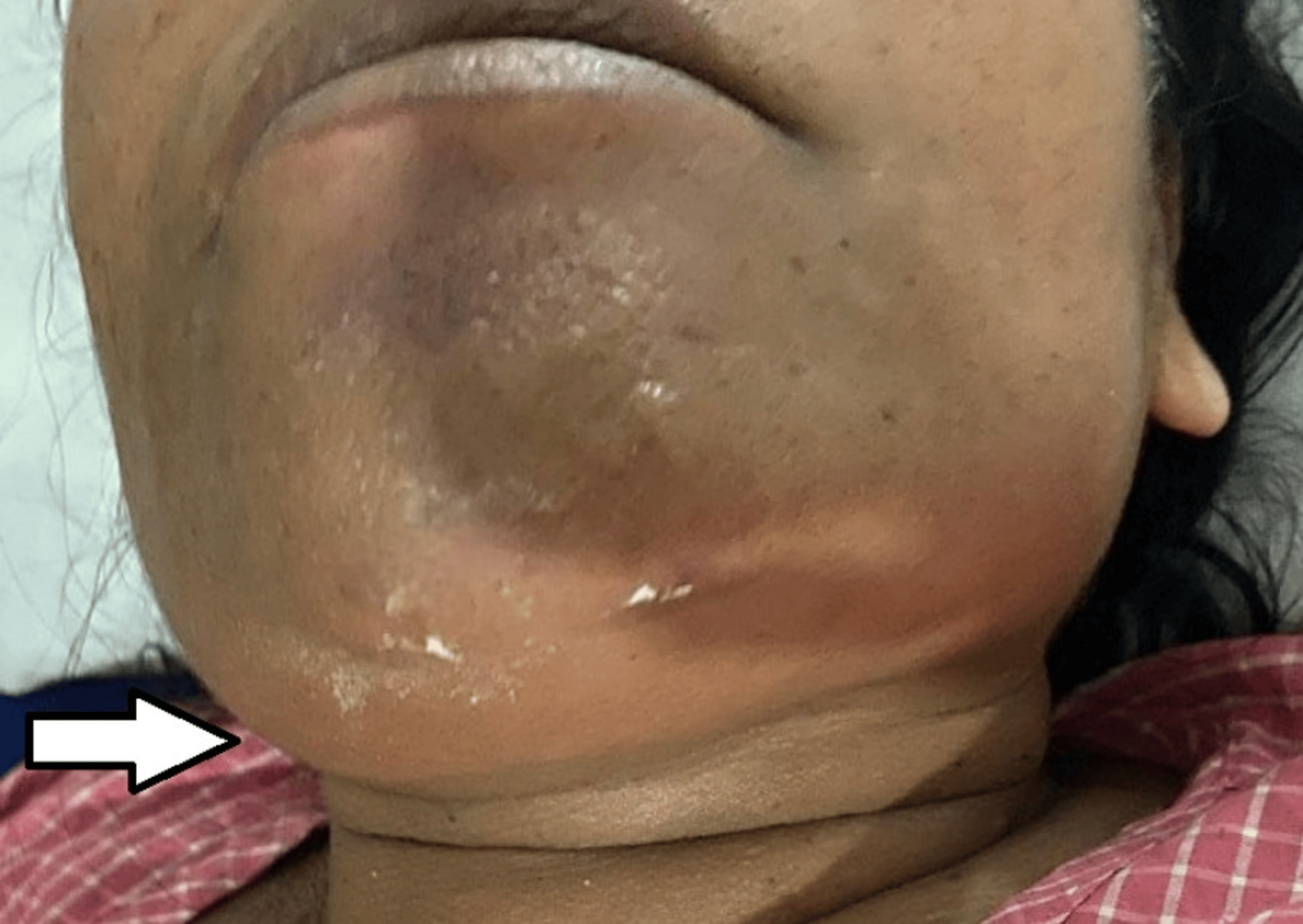
Preoperative image of the right submandibular swelling. Arrow marks swelling in the right submandibular region.

A 35-year-old hypertensive female patient came to the ENT OPD with complaints of swelling in the right side of her neck for the past seven months. Initially, the swelling was about the size of a lemon, which progressed to pear-sized. There was no associated pain, redness, fever, or discharge from the swelling. She also did not have complaints of any compressive symptoms. The patient had a history of incision and drainage in the same region four years ago for an abscess. On clinical examination, a solitary, ovoid swelling was present in the right submandibular region (level 1b), measuring 5 x 3 cm. On palpation, the swelling was cystic in consistency, non-tender, and there was no local rise in temperature. The skin over the swelling was normal. The rest of the ENT examination was normal (Figure 1).

Clinically, a provisional diagnosis of a cystic swelling of the right submandibular gland was made. Ultrasonography of the neck was performed, and it revealed an irregular hypoechoic collection of 33 x 5 x 33 mm, deep to the submandibular gland laterally from the sternocleidomastoid reaching up to midline medially, with few incomplete septations and a fat plane between the gland and collection, which was suggestive of abscess/infective collection.

**Figure 2:**
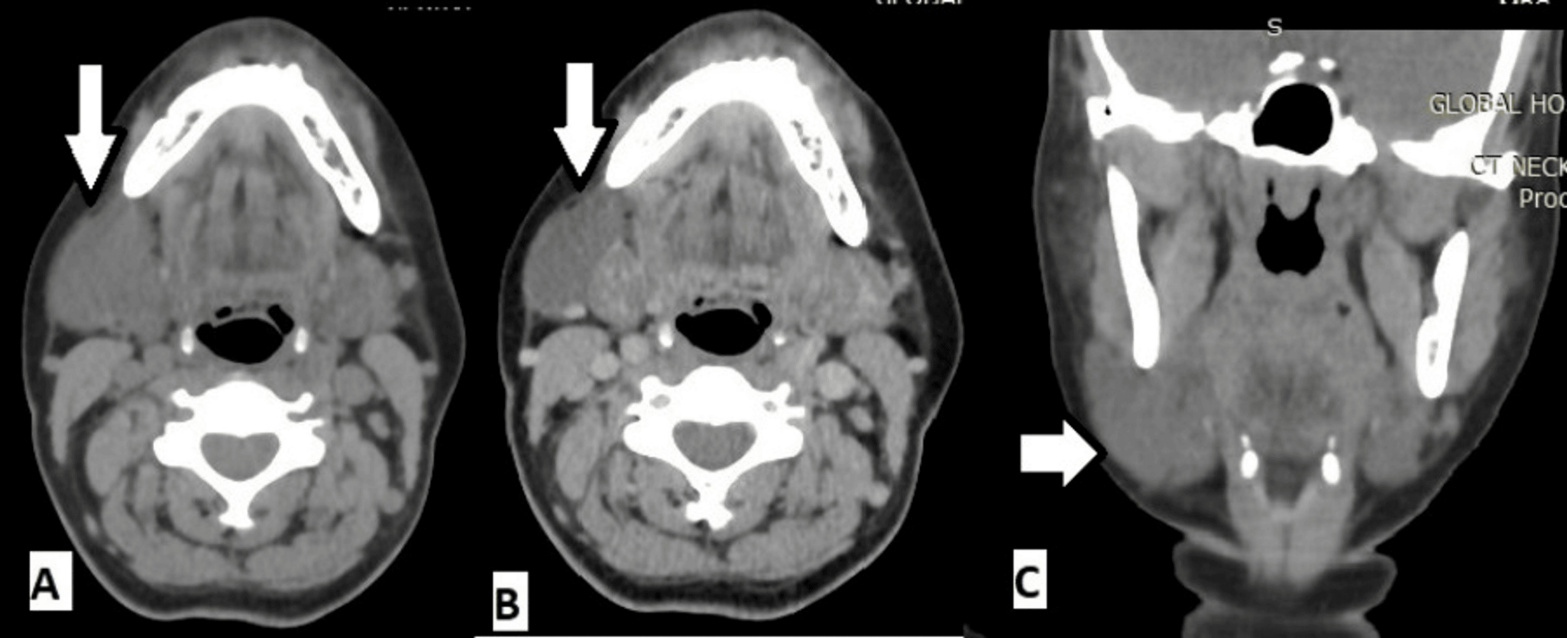
Contrast-enhanced computed tomography showing cystic lesion in the right submandibular region. (A) and (B) Axial cuts showing cystic lesion in the right submandibular lesion marked with the white solid arrow. (C) Coronal cut showing cystic lesion in the right submandibular lesion marked with the white solid arrow.

Contrast-enhanced computed tomography of the neck revealed a well-defined cystic lesion or walled-off collection along the anterolateral aspect of the gland, suggesting a branchial cleft cyst (Figure 2).

**Figure 3:**
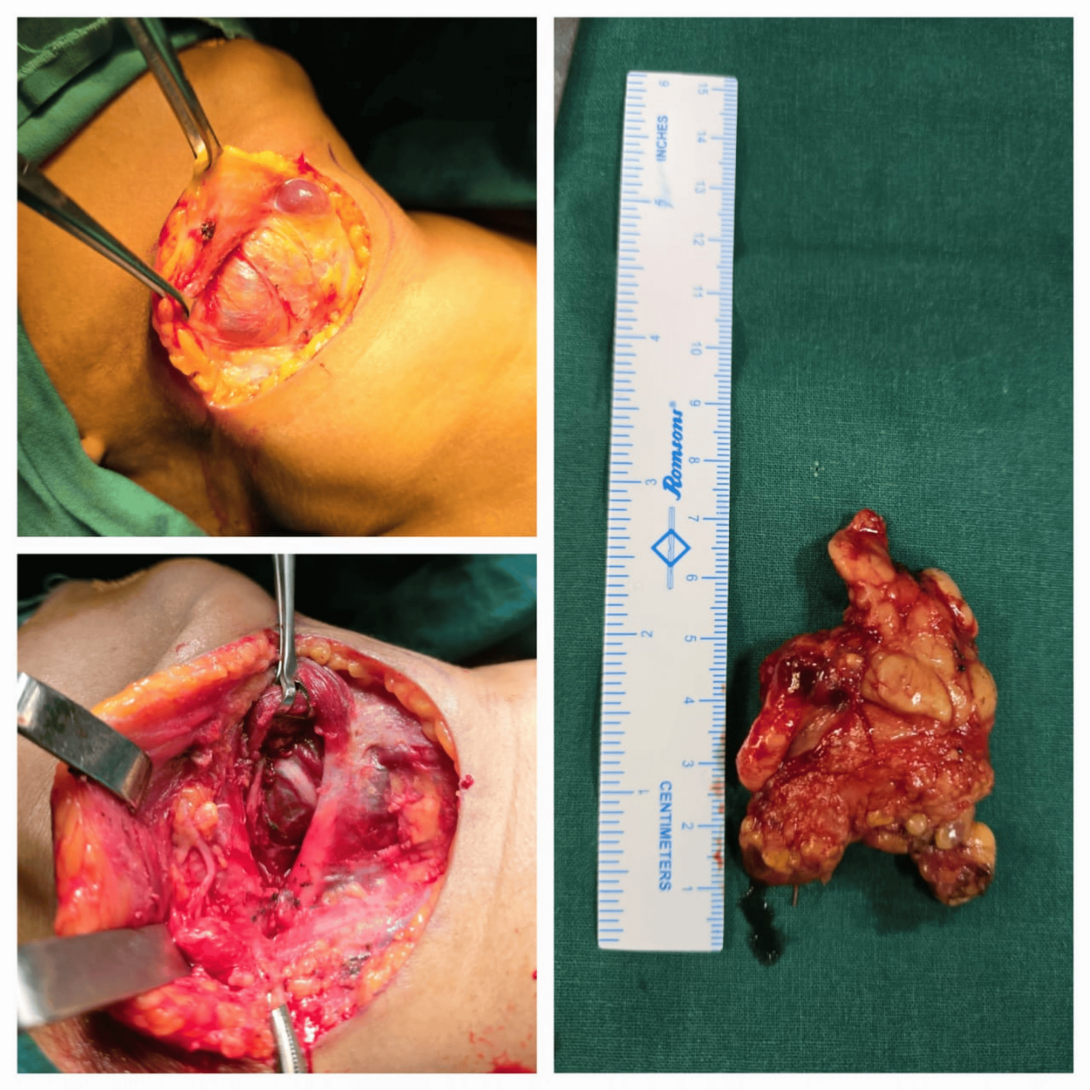
Intraoperative images.

The patient was planned for a complete submandibular gland excision under general anesthesia with all relevant consents. The right marginal mandibular nerve was preserved, and the Whartons duct was ligated. The procedure was uneventful (Figure 3).

**Figure 4:**
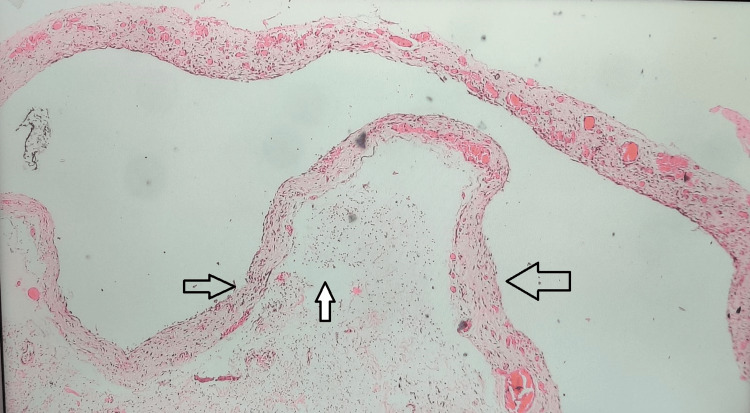
Histopathological image suggestive of cavernous lymphangioma. The black arrow points towards a cystic space lined by flattened epithelium, consistent with the appearance of dilated lymphatic vessels. The solid black arrow with a white fill points towards lymphoid aggregates, which are typically seen in the stroma of lymphangiomas.

Histopathological examination of the specimen revealed cavernous lymphangioma (Figure 4).

**Figure 5:**
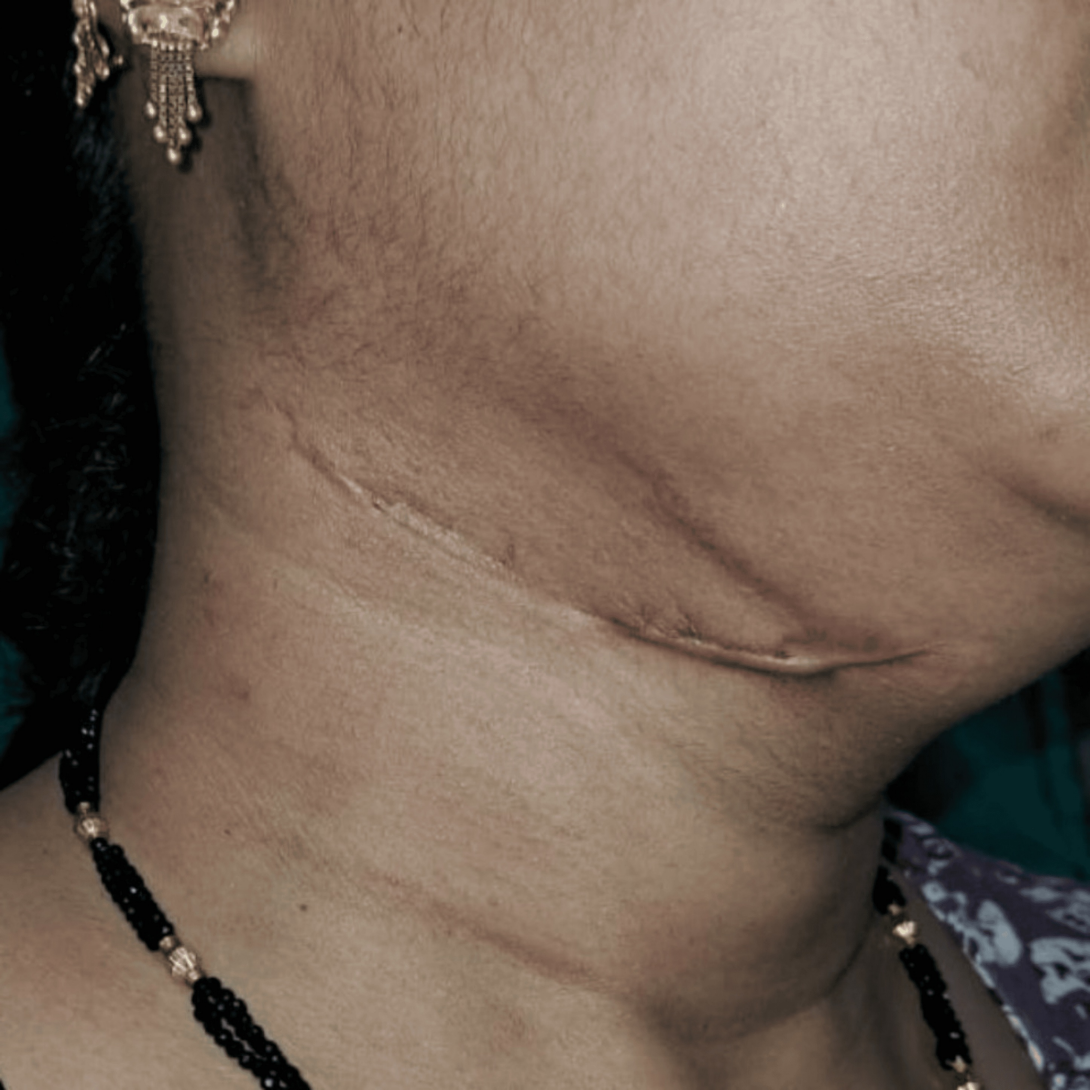
Postoperative image on three weeks follow-up.

The patient made satisfactory progress on follow-up after three weeks with no postoperative complications (Figure 5).

## Discussion

Lymphangiomas are hamartomatous lesions of lymphatic vessels that show a predilection for the head and neck regions. They arise due to developmental anomalies in the lymphatic pathway, usually occurring during the sixth week of gestation. Approximately 50% of cases are present at birth, with around 90% manifesting by the age of two years [7,8].

Three hypotheses have been suggested to elucidate the etiology of this anomaly. The first one suggests that a blockage in the typical development of primitive lymphatic channels takes place during embryonic development. The second theory says that the primitive lymphatic sac fails to connect with the venous system. The third hypothesis states that the lymphatic tissue is misplaced during embryonic development, leading to its presence in incorrect locations [1].

Lymphangiomas in the cervical region are infrequent, benign congenital anomalies primarily detected in childhood. These anomalies are uncommon in adults and predominantly manifest in the posterior cervical triangle (75% of cases), followed by the submandibular area (20%), and occasionally in various intra-abdominal locations. They are classified based on vessel caliber into capillary (in subcutaneous tissues), cavernous (in the oral cavity), and cystic (in the cervical region, termed cystic hygromas) [6].

Clinical manifestations are location-dependent, often presenting as a painless mass that progressively enlarges, although symptoms related to compression, such as dysphagia or dyspnea, may arise. Typically, the mass is soft, non-tender, and ill-defined. In adults, cosmetic appearance is a major concern.

Computed tomography serves as the primary diagnostic modality, aiding in surgical planning. Lymphangiomas typically manifest as cystic lesions that are multiloculated on a CT scan. In T2-weighted MRI images, they are isointense to cerebrospinal fluid, while their appearance on T1-weighted images varies due to differing protein concentrations [1]. Ultrasound examinations may reveal a hypoechoic tumor with trabeculae, which could indicate its proximity to neighboring structures.

Complete excision remains the cornerstone of treatment, especially for encapsulated lesions [9]. In our case, preoperative CT imaging facilitated precise surgical planning, ensuring the preservation of adjacent vital structures. Successful management of recurrent cases involved complete resection with careful clipping of lymphatic vessels. In cases where tumors are spreading along vital structures and complete surgical removal is not feasible, alternative treatments are considered. These may include procedures such as chemical drug sclerosis, sclerotherapy with intralesional bleomycin, radiotherapy, video-assisted surgical resection, or laser therapy. Sclerosis and laser therapy are commonly favored options, especially in pediatric cases [3,10]. In the past, radiotherapy has been effectively employed, but its use in children is limited due to potential risks [11]. Berrada et al. conducted a retrospective study on 17 patients and found that complete resection was possible only in 12 patients [12]. Kumar et al. conducted a study on 35 lymphangioma patients, and they found that the neck was the most common site of involvement, with an excellent response in seven patients, a good response in 26 patients, and a poor response in two patients [13]. Fasching et al. conducted a study in 37 lymphangioma patients and treated them with OK-432 (Picibanil) and noted that the response to treatment was excellent or good in 32 patients; two patients had a medium response, and three did not show any response [14]. In adults, these therapies are typically reserved for treating relapses or as adjuvant therapy following surgery. The most frequently reported complications are infection, hemorrhage, hematoma, seroma, and injury to the nerves in the region. Overall, meticulous surgical planning and execution contributed to favorable postoperative outcomes for our patients.

## Conclusions

Lymphangioma in the cervical region in adults is a rare, benign malformation typically associated with prior cervical infections, trauma, or previous neck surgeries. Diagnostic modalities, such as axial computed tomography or magnetic resonance imaging of the neck, are preferred for accurate evaluation and surgical planning. Presently, surgical excision stands as the preferred therapeutic approach due to its minimal mortality and low recurrence rate. The etiological factors underlying the occurrence and progression of cervical lymphangioma in adults remain ambiguous, necessitating further elucidation of the underlying mechanisms.
